# Air separation with graphene mediated by nanowindow-rim concerted motion

**DOI:** 10.1038/s41467-018-04224-6

**Published:** 2018-05-04

**Authors:** Fernando Vallejos-Burgos, François-Xavier Coudert, Katsumi Kaneko

**Affiliations:** 1 Center for Energy and Environmental Science, Shinshu University, Nagano, 380-8553 Japan; 2 Chimie ParisTech, PSL University, CNRS, Institut de Recherche de Chimie Paris, Paris, 75005 France

## Abstract

Nanoscale windows in graphene (nanowindows) have the ability to switch between open and closed states, allowing them to become selective, fast, and energy-efficient membranes for molecular separations. These special pores, or nanowindows, are not electrically neutral due to passivation of the carbon edges under ambient conditions, becoming flexible atomic frameworks with functional groups along their rims. Through computer simulations of oxygen, nitrogen, and argon permeation, here we reveal the remarkable nanowindow behavior at the atomic scale: flexible nanowindows have a thousand times higher permeability than conventional membranes and at least twice their selectivity for oxygen/nitrogen separation. Also, weakly interacting functional groups open or close the nanowindow with their thermal vibrations to selectively control permeation. This selective fast permeation of oxygen, nitrogen, and argon in very restricted nanowindows suggests alternatives for future air separation membranes.

## Abstract (web summary)

Graphene with nanowindows can have 1000 times higher permeability and four times the selectivity for air separation than conventional membranes, Vallejos-Burgos et al. reveal by molecular simulation, due to flexibility at the nanoscale and thermal vibrations of the nanowindows' functional groups.

## Introduction

Separation processes comprise 40–70% of both capital and operating costs in modern industries^1^; increasing their efficiency then becomes critical. Separations based on distillation account for almost 8% of the total US energy consumption^2^, thus having a very large carbon footprint due to carbon dioxide (CO_2_) production^3,4^. The path to a sustainable future requires not only to improve the efficiency of the distillation processes but also to develop alternative separation technologies. Membrane-based techniques offer many benefits over conventional technologies based on phase transitions^5^. These benefits are a smaller equipment footprint, reduced mechanical complexity, and the requirement of 90% less energy than distillation^2^, resulting in much lower CO_2_ emissions. After mastering membrane technologies, separations based on phase changes will become obsolete.

Graphene is one of the recent superstars in the materials field and keeps growing in importance. With its synthesis processes continuously improving, we expect to achieve large-scale production with an exceptionally low concentration of defects. Due to its one-atom thickness, robustness, chemical stability, and ability to be converted into a sieve, one of its most important applications will be its usage as a membrane^6–9^.

Distillation is widely employed in the air separation industry to obtain high purity N_2_, O_2_, and noble gases, with significant amounts of energy spent on phase transitions^10^. Efficient membrane-based technologies have the potential to decrease the air separation industry’s energy demand and reach higher product selectivities.

As pristine graphene sheets are impermeable even to the smallest gases such as helium^11^, it is thus necessary to introduce nanoscale windows (nanowindows) to transport and separate molecules. These nanowindows surpass conventional membranes, as their single-atom-thick wall provides almost negligible pore transport resistance, exhibiting ultra-fast molecular permeation^12^. We prefer the term nanowindows^13,14^ instead of nanopores, as the latter is a nanospace of deep-potential ready-to-adsorb molecules, whereas nanowindows are atomically thick hole defects in a single-layer structure with the ability to open and close. Many methods can create nanowindows in graphene, e.g., ion bombardment^15^, template-synthesized mesh^16^, and simple high-temperature oxidation^17^.

The graphene nanowindow and especially its rim chemistry, featuring functionalized C edges, become critical factors determining the permeation rate and the selectivity of graphene membranes. The related computational studies in the literature, addressing the calculation of selectivity and permeation rates through nanowindows, are just as realistic as the membrane models employed. Extreme cases of an idealized nanowindow in graphene^18–23^ consist of removing some predetermined C atoms and modeling the remaining framework only through dispersion interactions. Although this can estimate the order of magnitude of permeation energies, it fails to estimate permeation rates due to their exponential relationship to energy. Moreover, most existing studies disregard the dynamic nature of the nanowindow and its rim chemistry, thus failing to predict the unprecedented permeation mechanism of graphene membranes.

Nanowindows are dynamic, as their rims vibrate and relax: the relaxation of a nanowindow in an annular polyaromatic molecule decreased two to five times the permeation energy barrier, depending on the permeating molecule^24^. Reducing the graphene framework to a non-periodic polyaromatic molecule also overemphasizes the role of relaxation, as the whole periodic framework is stiffer than the isolated molecule and gives rise to phonon vibrations. In the highly active metal organic framework (MOF) area, it has been demonstrated that the flexibility induced by adsorbed molecules also changes the structure of an MOF^25,26^. Incorporating flexibility into the models becomes crucial, as it is concurrent to molecular permeation.

A correct description of the rim chemistry of graphene nanowindows is then essential for practical atomistic simulations aiming to design highly selective membranes, because, as we will show, the presence of heteroatoms and defects induce an electric field around the nanowindow rim, which interacts with some permeating species. This important factor, known to have a big role in adsorption, e.g., in the case of zeolites^27^, is unaccounted for in idealized nanowindows. Moreover, functional groups such as hydroxyl, carboxyl, and carbonyl possess several orientations due to libration or torsion with respect to the nanowindow. Their dynamic orientations alter the nanowindow shape^28^ and effective size, which affects the permeation mechanism and its selectivity. Consideration of these functional groups has been limited mainly to stacked graphene oxides^7^.

Describing permeation only as dictated by geometric factors, such as the nanowindow size, becomes inaccurate if we consider that, owing to a thermal distribution of kinetic energies, molecules permeate even through a nanowindow smaller than its effective size. The geometrical size and shape of the nanowindow and the permeating molecule, and the chemistry and motion of the nanowindow rim taken altogether govern molecular permeation. In particular, the role of a concerted motion of nanowindow rims and their functional groups must be elucidated, because the dynamic motion of the nanowindow rim and the partial charge distribution produced by the heteroatoms give rise to molecular-recognition-type penetration, which can be applied to highly efficient molecular separation.

Taking on the challenge of separating molecules with similar sizes, but different interactions, gives information about their interactions with nanowindow rims. We choose the main air components, as their separation is industrially relevant and they exemplify different types of molecular interactions (dispersive and electrostatic), to discuss the impact of the nanowindow rims’s assistance^29,30^. Our computer simulations show that realistic nanowindows can achieve four times the selectivity of experimentally reported membranes at a thousand times higher rate.

## Results

### Effective sizes and polar states of air molecules

In confined nanospaces, defining molecular dimension depending on the orientation that the molecule can adopt inside the restricted space is most appropriate. To permeate through a narrow nanowindow (see a nanowindow definition in Supplementary Fig. 1), two of the three orientation-dependent sizes will be constrained, and then the largest constrained dimension or second minimum dimension (MIN-2) in this case determines an effective molecular size for each gas molecule, which are 2.99, 3.05, and 3.63 Å for O_2_, N_2_, and Ar, respectively^31^. A practical nanowindow sieve for air separation should range near these dimensions.

N_2_ and, to a lesser extent, O_2_ possess quadrupole moments that interact with other charges, such as the functional groups in the nanowindow rim. In contrast, Ar lacks electrostatic interactions. These differences create opportunities for a separation based on selective interactions.

### Permeation rate through realistic nanowindows

With respect to molecular dynamics (MD) simulations, literature contains various cases of simplified nanowindows, e.g., without any heteroatom^18,21,23^, a rigid framework^20–22^, or too idealized rim symmetries^18,19^. To improve the phenomenological description, we created more realistic and feasible nanowindows by including functional groups and a totally flexible graphene framework.

**Fig. 1:**
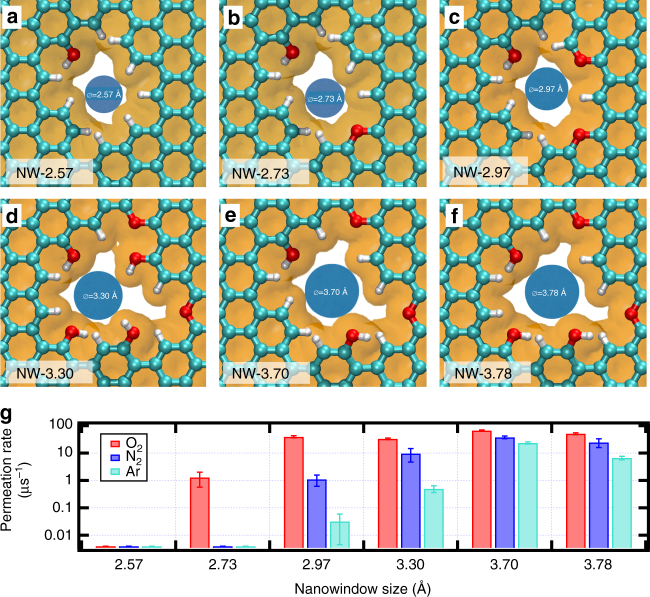
Some quantum chemically allowed models of nanowindows. Each nanowindow is denoted as NW-*d* where *d* is van der Waals diameter of the opening in Å, represented by brown shadings. Cyan, red and white atoms are C, O, and H, respectively. Resulting sizes in Å were **a** 2.57; **b** 2.73; **c** 2.97; **d** 3.30; **e** 3.70; and **f** 3.78. **g** Permeation rate constants evaluated fitting the average of many MD runs in a  canonical ensemble to a first order model. Temperatures are 90, 77, and 87 K for O_2_, N_2_, and Ar, respectively. Permeation rates barely shown indicate that no permeation events were observed during our simulations. Error bars represent uncertainty ± 1 SD. There is highly selective O_2_ permeation through a nanowindow smaller than the O_2_

Six different nanowindow models were designed (see Fig. 1a–f) with a size similar to the permeating molecules. Notation for each nanowindow is NW-*d*, where *d* is the aperture size ranging from 2.57 to 3.78 Å. We followed three considerations in building the nanowindows: first, our nanowindows are created by oxidation in air and thus the C-edges are passivated with H or O atoms. Passivation of the free C-edges results in a MJ mol^−1^ order of decrease in system energy (see Supplementary Table 1). Second, the oxygen content increases with oxidation^21,32^ with the heteroatoms existing mainly as ubiquitous hydrogen^33^ (R–H), phenol^34–37^ (R–OH), and ether^37–39^ (R–O–R’); and third, the aperture sizes are between 2.57 and 3.78 Å, which reasonably encloses the range of molecular sizes of interest in air separation.

Permeation rates were evaluated for each nanowindow from MD simulations in the canonical ensemble. The simulation cell consists of two compartments separated by graphene with a nanowindow, where initially one compartment is filled with gas. Rates reported are the rate constants considering first-order permeation kinetics (see derivation in Supplementary Note 1 and Supplementary Fig. 2). The permeation steps and mechanisms resulting in a nanowindow crossing were already analyzed in several excellent papers^19,40–42^. Briefly, for a permeation event to occur, the molecule first needs to be transported from the gas phase to the adsorbed state near a nanowindow in the graphene basal plane. Then, it needs to leave the basal plane and move to the top of the nanowindow, in an incipient permeation position. Finally, it propagates through the nanowindow (see all steps in Supplementary Fig. 3) and desorbs on the other side of the membrane. In general, the gas permeation rate constants (see Fig. 1g) decrease with nanowindow size. Due to their large size, NW-3.78 and NW-3.70 have a high permeation rate and low separation selectivity. Narrowing the size to 3.30 Å decreases the Ar permeation rate constant by a factor of 50 and increases the N_2_/Ar selectivity to 20, evidencing a molecular sieving regime.

Deeper observation of the tendency in the permeation rate constants evidences some (surprising) facts. For example, O_2_ permeates faster through NW-2.97 than NW-3.30 and N_2_ permeates faster through NW-3.70 than NW-3.78. In both cases, this is due to the increased population of permeating molecules on top of the nanowindow (density profiles for the case of O_2_ are shown in Supplementary Fig. 4). O_2_ population on top of the nanowindow is five times larger in NW-2.97 than in NW-3.30; in the case of N_2_, it was 1.7 times larger. Finally, a faster Ar permeation rate of NW-3.70 compared with NW-3.78 occurred due to the lower system energy when Ar (MIN-2 size is 3.6 Å) is inside a non-sterically confined nanowindow: a calculation of energy profile showed intra-nanowindow maximum energies of an Ar atom of − 953 K and − 708 K for NW-3.70 and NW-3.78, respectively. This energy difference readily explains the different permeation rates.

### Permeation rate and molecular size

Surprisingly, molecules permeate even if their effective size is larger than the nanowindow itself, as the high-energy (or transition) state occurs only temporarily and can be overcome by kinetic energy fluctuations of the incoming particle.

This is evident in the case of nanowindows NW-2.97 and NW-3.30 where even Ar permeates, at a size (MIN-2 size is 3.63 Å) at least 10%–22% larger than the nanowindow. In these cases, the permeation transition state lasts less than a picosecond in our simulations at 87 K. This phenomenon is reminiscent of MOF: ZIF-8 with its extraordinary framework flexibility, capable of adsorbing hydrocarbons larger than its nominal aperture size^43^.

**Fig. 2:**
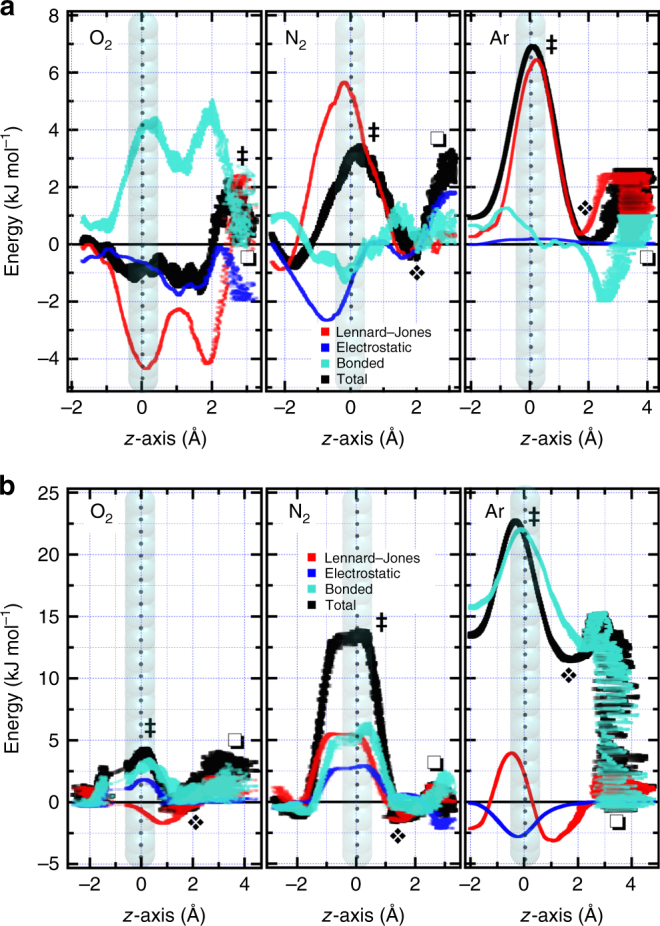
Enthalpy contributions to permeation through nanowindows. **a**, **b** Permeation energy barriers of O_2_ (90 K), N_2_ (77 K), and Ar (87 K) through NW-3.30 **a** and NW-2.97 **b**. The *z*-axis is perpendicular to the graphene layer, located at *z* = 0. Total energy (black line) sums the contributions from Lennard–Jones (red), electrostatic (blue), and bonded interactions (turquoise). Bonded interaction energy considers bonds, angles, dihedrals, and impropers. Square, diamond, and double dagger symbols represent adsorption on the basal plane, adsorption on top of the nanowindow, and a high-energy state across the graphene plane, respectively

A reaction pathway for a small gas molecule being pulled through a nanowindow (see Fig. 2) has generally two local energy minima and a single transition state. Local minima occur when the molecule is adsorbed on the basal plane at *z* equals 3.3 Å or on top of the nanowindow at around 2 Å. The high-energy state occurs in the in-graphene plane at *z*-axis is 0 Å when the molecule squeezes through the nanowindow rim. O_2_ freely permeates through nanowindow NW-3.30 due to its smaller size, lacking an in-graphene plane transition state (Fig. 2a). The enthalpy barriers for crossing nanowindows of similar size as the permeating molecule are in the order of 3–6 kJ mol^−1^. For Ar, the main contributor to the total enthalpy at the transition state is dispersion interaction due to its repulsion term, whereas for N_2_ this repulsion is counterbalanced by a strong electrostatic stabilization.

When permeating a narrower nanowindow (see Fig. 2b), O_2_ and N_2_ face one energetic barrier of 4 and 13 kJ mol^−1^, respectively. However, Ar exhibits super-slow permeation due to two consecutive energy barriers that deform the functional groups when displaced from the basal plane to the top of the nanowindow, and then another deformation by crossing the graphene layer.

### Rim heteroatoms induce a strong electrostatic field

**Fig. 3:**
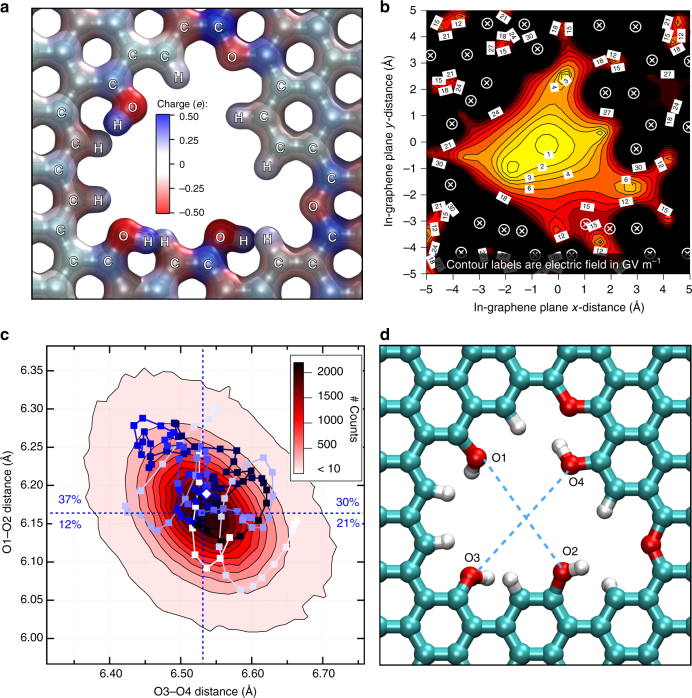
Large electrostatic field and breathing of two nanowindows. **a** Partial charges in NW-3.78 nanowindow calculated according to MK scheme. **b** Contour plot for in-graphene plane magnitude of the electric field in GV m^−1^ calculated from partial charges shown in **a** for NW-3.78. Circles with crosses indicate position of atoms. **c** 2D histogram contour of the distances between two pairs of O atoms on opposite sides of the rim during an MD simulation in NW-3.30. Vibration is asymmetric with a Pearson correlation coefficient between O1–O2 and O3–O4 of − 0.38. The blue-gradient path corresponds to the distances when a N_2_ molecule permeated through the nanowindow. Darker marks correspond to N_2_-center of mass closer to the nanowindow center in the *z*-direction. **d** Atomic numbering used to calculate lengths in **c**. Cyan, red and white atoms are C, O, and H, respectively

The different electronegativities of the H or O atoms bonded to C at the rim of the nanowindow, together with the addition of defects in the graphene network, induce heterogeneity in the electronic density of the nanowindow rim atoms. These partial charges (see Fig. 3a) along the rim create an electrostatic field around the nanowindow of GV m^−1^ magnitude (Fig. 3b). This interacts differently with the quadrupole moments of O_2_ and N_2_.

The effect of this large electric field is evident by comparing electrostatic potential energies of air molecules while permeating the nanowindow (see Fig. 2a), as O_2_, N_2_, and Ar interactions are weak, strong, and non-existent, respectively. In the case of N_2_, the dispersion repulsion, 5.6 kJ mol^−1^, is strongly counteracted by an electrostatic stabilization of − 2.7 kJ mol^−1^, resulting in a total energy requirement of only 3.1 kJ mol^−1^. However, in the case of Ar, the main contribution to total energy arises from its repulsion with nanowindow rim atoms.

### Nanowindows breathe and relax

Graphene has phonon motion and local oscillations, which give rise to concerted vibrations in the nanowindow rim (see Supplementary Movie 1). These vibrations alter the effective size and shape of a nanowindow, thus determining its permeation properties. The distribution of these concerted vibrations at the rim can be visualized via a two-dimensional contour histogram of the distances between opposing O atoms during a simulation (see Fig. 3c–d). Although a high concentration of distances is at the center of the plot, thermal energy creates fluctuations of these atomic distances in about 0.1 Å. The Lennard–Jones (LJ) energy difference when an Ar atom approaches four O atoms from 3.30 Å to 3.25 Å shows that this concerted motion indeed involves a considerable energy penalty of 0.5 kJ mol^−1^.

The O–O pair distances were followed while N_2_ permeated through the nanowindow (path in Fig. 3c). As bond vibrations are around 10 times faster than permeation, there is a large fluctuation of interatomic distances. Most permeation occurred when the shorter O–O distance was above its average, due to the intuitive fact that the nanowindow vibration is sterically constrained when a molecule is inside. The gas permeation then becomes concerted to the nanowindow vibration, allowing the rim to enlarge due to a molecular crossing event. This is clear by comparing against a rigid nanowindow (see in Table 1 the rigid nanowindows labelled NW-*n*C, with *n* the number of C atoms removed) where frameworks with similar selectivity have much lower permeation rates. Such coherent vibration is very similar to that observed in the framework dynamics of small-pore zeolites, where it is called a “window breathing” mode, which significantly impacts guest diffusion and molecular sieving^44^. This asymmetrical nanowindow breathing mode is employed in the separation of molecules by shape.

**Table 1:** Nanowindows separate air much faster than conventional membranes

Membrane type	Rate (GPU)	Selectivity O_2_/N_2_	Temperature (K)	Reference
Graphene nanowindow NW-2.73	27,100	45	195	This work
Graphene nanowindow NW-2.97	278,000	7.3	195	This work
Graphene nanowindow NW-2.73	10,040	20	300	This work
Graphene nanowindow NW-2.97	60,500	5.0	300	This work
Graphene nanowindow NW-8C	1950	62	300	This work
Graphene nanowindow NW-10C	25,070	6.8	300	This work
Graphene nanowindow NW-13C	1,467,800	1.1	300	This work
Polysulfone hollow fiber	43	5.2	323	Kesting et al.^47^
Asymmetric polysulfone hollow fibers	8.8	5.8	297	Pesek and Koros^48^
6FDA-durene hollow fiber	64	3.20	298	Chung et al.^49^
6FDA-durene hollow fiber	1512	1.09	298	Chung et al.^49^
Matrimid 5218 polyimide hollow fiber	7	7.5	297	Clausi and Koros^50^
Matrimid 5218 polyimide hollow fiber	33.7	6.0	297	Clausi and Koros^50^
Modified polyphenylene oxide film	648	3.9	308	Sterescu et al.^51^
Polyimide hollow fiber	300	3	333	Liu et al.^52^
Polydimethylsiloxane hollow fiber	60	1.8	298	Prajapati et al.^53^
Polydimethylsiloxane hollow fiber	7	7	298	Prajapati et al.^53^

Selectivity of nanowindows is highly sensitive to rim vibration, as shown by its dependence on temperature. Gain in rate decreases selectivity. Graphene nanowindows are four orders of magnitude faster and more selective than conventional membranes

### Rotations of functional groups open or close nanowindows

He et al.^28^ demonstrated that nanowindows with negatively charged carboxylate groups present asymmetric permeation energy profiles at each side of the graphene wall. This was due to different ways that the carboxylate groups orient toward the graphene plane, creating different environments on each side of the graphene wall. Later, Drahushuk et al.^41^ experimentally found that nanowindow-rim bonds can be chemically rearranged, thus modifying the nanowindow shape and consequently the permeation barrier to achieve selective gas permeation. It is then required for computer simulations to sample all allowed configurations of the nanowindow by considering a flexible framework.

**Fig. 4:**
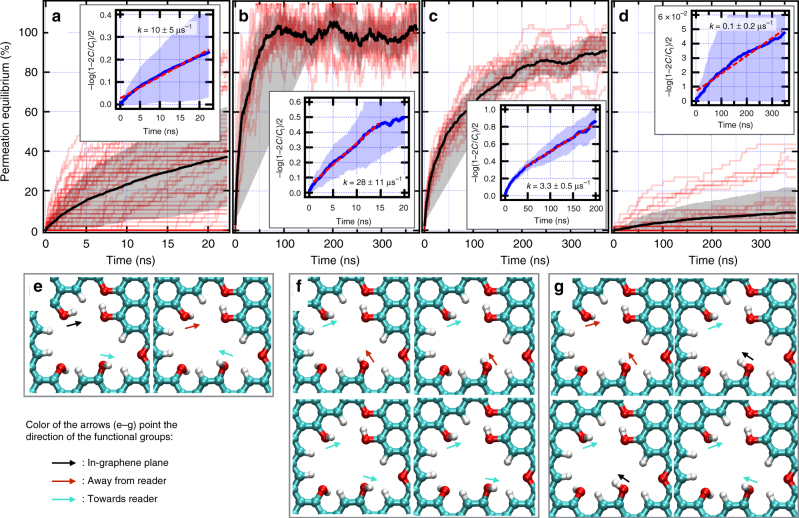
Effect of nanowindow dihedral rotation on N_2_ permeation rates. **a**–**d** Molecular dynamics results of N_2_ permeability at 77 K. Thin red lines are single runs, the thick black line is the average of all runs in red and the shaded area is standard deviation. The inset is the linearization of all data including a linear fit to the average. *k* is the permeation rate constant where uncertainty is 1 SD. Results are divided in **a** all results for a flexible nanowindow framework and for a rigid framework grouped in **b** fast, **c** medium, and **d** slow. **e**–**g** Show several representative orientations of functional groups for **b**, **c**, and **d** regimes, respectively. Colored arrows indicate the orientation of O(1–2)–H bond. Cyan, red, and white atoms are C, O, and H, respectively. All these results correspond to NW-3.30

Similar to these out-of-plane configurations, other out-of-plane functional groups dynamically switch their orientation. We analyzed the effect of dihedral orientation of hydroxyl groups in the nanowindow rim (e.g., O1–O4 in Fig. 3d). A set of simulations run for N_2_ permeation through the flexible NW-3.30 framework (see Fig. 4a) show large-scale fluctuations, with a large spread in permeability rate constants (*k* = 10 ± 5 µs^–1^). During all our results presented so far, the full system was allowed to vibrate. The H atoms (which only electrostatically interact with N_2_ and O_2_) in the hydroxyl groups are free to rotate through H–O–C–C dihedrals, temporarily locking the H in local energy minima configurations. In contrast, starting the simulations as fully flexible and then quenching the nanowindow framework rigid evidences the importance of these configurations. It allowed identifying three distinct rate regimes of permeation separated by orders of magnitude. A fast permeation regime (*k* = 28 ± 11 µs^–1^, Fig. 4b) occurs when the OH atom pairs point toward opposite sides of the graphene plane (Fig. 4e, with O-atoms notation in Fig. 3d), allowing a larger nanowindow space to be opened for the permeating N_2_. In all these cases, H–O2 pointed toward the reader, creating a favorable environment for permeation. In the moderate rate permeation regime (*k* = 3.3 ± 0.5 µs^−1^, Fig. 4c), the OH atom pairs point in opposite out-of-graphene-plane sides and converging graphene-plane direction or same out-of-graphene-plane side but diverging graphene-plane directions (see Fig. 4f). Finally, in the slow rate permeation regime (*k* = 0.1 ± 0.2 µs^−1^, Fig. 4d), both atom pairs choke the nanowindow by pointing and also converging in the same direction (see Fig. 4g). Changes in O3–H hydroxyl group orientation had no effect on permeation.

Then, through torsion of functional groups H–O1 and H–O2, the nanowindow electrostatically (as H is just modeled as a point charge) closes to N_2_. This shows that the OH group position closes the nanowindow to electrostatically interacting molecules, showing strong potential for enhanced separations by including other functional groups in the rim.

**Fig. 5:**
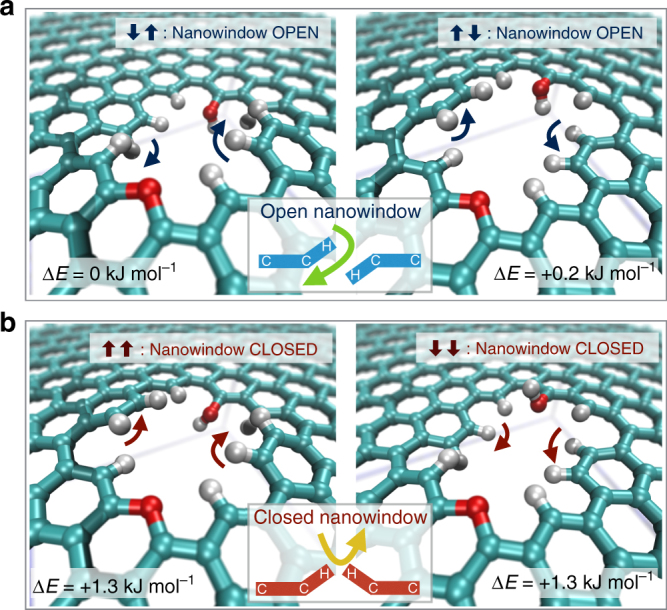
Functional group flapping mechanism in a small nanowindow. The nanowindow can be in an open or closed states for allowing O_2_ permeation. **a** Open states. Pairs of hydrogen point in different directions. **b** Closed states. Pairs of hydrogen point in the same direction. Arrows indicate the directions where the H-pairs point. Energy difference is respective to the structure **a**, left. Cyan, red, and white atoms are C, O, and H, respectively. Insets illustrate the equivalence between flapping movement and the opening/closing of gates. These figures correspond to O_2_ permeation at 90 K through NW-2.73

As O_2_ is smaller than N_2_, it is natural to require a smaller nanowindow to observe a strong effect of the rim functional groups movement on permeation. This sieving has to occur where the molecular crossing is constricted by the rim, i.e., NW-2.73 and smaller for O_2_ (notice the decrease in O_2_ permeation rates in Fig. 1). As in the previous case with N_2_, permeation with the graphene framework locked rigid in different energy minima, showing the importance of the functional group vibration in even narrower nanowindows. Two different O_2_ permeation cases occur in NW-2.73 depending on the mutual orientation of the opposing armchair hydrogen pairs in the nanowindow rim: (i) an open regime (see Fig. 5a, rate constant 1.8 µs^−1^) when H-pairs flap away from the graphene layer in opposing directions, allowing permeation of O_2_ and (ii) an impermeable regime (see Fig. 5b, rate constant less than 0.001 µs^−1^) when both H-pairs flap toward the same direction and close the nanowindow. Open positions are slightly more favorable (∆*E* = − 1.3 kJ mol^−1^), which is confirmed by their number of occurrences. This flapping movement is correctly sampled in the flexible framework, as its rate constant contains contributions from both closed and open states of the nanowindow.

### Narrow nanowindows separate air ultra-fast and selectively

Selectivity was first evaluated with the ratio of single gas permeation. A very oxygen-selective and fast-permeating nanowindow is NW-2.97: its permeation rate constant is 47 µs^−1^, which translates into 600 m^3^ STP min^−1^ m^−2^ and a selectivity larger than 50 for O_2_:N_2_ separation (and 1500 for O_2_:Ar). Narrower membranes, such as NW-2.73, allow slow permeation of O_2_, while totally blocking N_2_ and Ar in the 200 ns timescale of our simulations.

**Fig. 6:**
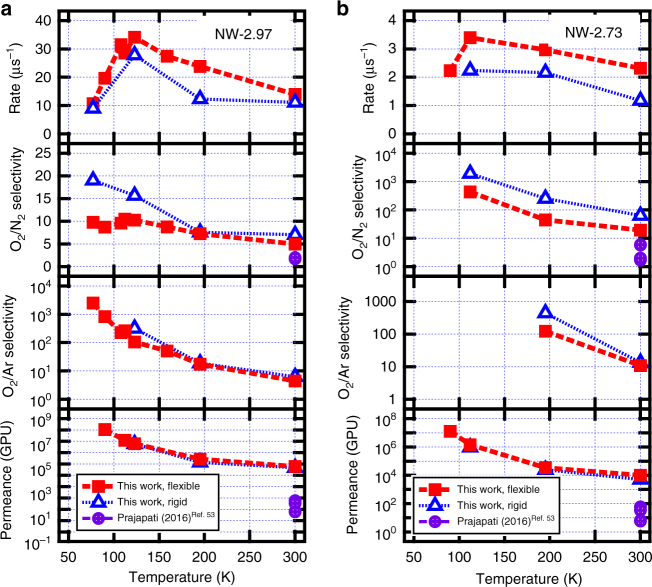
Nanowindows NW-2.97 and NW-2.73 effectively separate air. Temperature dependence for the various parameters in **a** NW-2.97 and **b** NW-2.73. Optimum rates occur at a temperature with both high kinetic energy and high adsorption amounts. For calculation of GPU (10^−6^ cm^3^ (STP) cm^−2^ s^−1^ cm Hg^−1^), the ideal gas pressure was estimated from the density of the gas far from the surface where it behaves as bulk

In following more realistic experiments, a model mixture of air (O_2_:N_2_:Ar = 1:1:1) was easily separated at different temperatures through NW-2.97 (see Fig. 6a). O_2_:N_2_ selectivity does not change much with temperature, indicating that both molecules can easily cross the membrane. However, O_2_:Ar selectivity is very sensitive to temperature, reaching extremely high selectivities at cryogenic temperatures. Modeling the framework as rigid (blue triangles in Fig. 6) decreases permeation rate due to the constraint in the nanowindow vibrations but slightly raises selectivity.

A narrower nanowindow (see NW-2.73 in Fig. 6b) is much more attractive for separation purposes. Even though permeation rates are lower compared to NW-2.97, its selectivity to O_2_ is higher and it dramatically increases after lowering the temperature. The monotonic decrease of selectivity with rising temperature is a consequence of it being the quotient of the rates of two activated processes. Similarly, the increase of permeance with rising temperature is due to the large changes in gas pressure (the simulation ensemble was canonical) with temperature: in these simulations or air permeation, the calculated pressure was 0.05, 0.7, 1.3, 25, and 67 bar at *T* = 90, 112, 122, 195, and 300 K, respectively. Above 155 K, all these gases are already supercritical.

A more thorough comparison with experimental membranes (see Table 1) shows some crucial points. First, increase in selectivity comes at the cost of decreased permeability. Second, commercial polymers, such as polysulfone, polycarbonate, and polyimides, can only reach permeation rate selectivities of about 6 for O_2_:N_2_^45,46^. And third, even the best polymer membranes, including those with a mixed matrix, rarely surpass an O_2_:N_2_ selectivity above 10^45,47–53^. This can only be obtained at permeability rates many orders of magnitude lower than our graphene nanowindows. This breakthrough in separation membranes becomes clear in a Robeson plot (see Supplementary Fig. 5) where the upper bound is extended by two orders of magnitude if we use our nanowindows at low temperature. This immense potential of ultra-fast separation with graphene nanowindows can be exploited in multistage setups at low temperature, where purity can be quickly raised after each stage.

## Discussion

The behavior of graphene nanowindows of atomic sizes is highly dynamic and dependent on its rim functionalization. Nanowindow breathing, relaxation, rotation of bonds, and large electrostatic fields enable nanowindows to open or close to molecular permeation. Simplified nanowindow models, which ignore the distribution of charges or flexibility of the framework, cannot describe these mechanisms. We showed that high separation selectivities, and especially ultra-fast permeation rates, a thousand times larger than current membranes is achieved and thus encourage further development in description and development of atomically thin membranes for molecular separation.

## Methods

### Nanowindow construction

Graphene nanowindows were modeled by removing carbon atoms from the center of a pristine graphene layer and passivating the exposed edges with –H, –OH, or C–O–C terminations. The nanowindow structure was optimized with MOPAC2016^54^ and the PM7^55^ method to report the size of the nanowindow as the radius of the largest sphere that fits in the nanowindow, when it just contacts the van der Waals radii of the rim edge atoms.

### Molecular dynamics

MD simulations were run using HOOMD^56–58^ code, version 1.3.3. The whole graphene nanowindow framework was considered fully flexible with partial charges along the nanowindow rim. Ar was modeled as a single center LJ particle, whereas O_2_ and N_2_ were modeled as rigid bodies consisting of two LJ particles plus three point charges, to take into account their quadrupole moments. A comparison of the charges considered in our models against those calculated by density functional theory (DFT) is shown in Supplementary Fig. 6. Bonded and non-bonded force field parameters are listed in Supplementary Tables 2 and 3. Vibrational frequencies obtained for a hydroxyl-coronene molecule with this force field are compared against those of a high-level DFT calculation in Supplementary Fig. 7. The vibrations playing a key role in permeability, such as –H switching sides of the graphene layer, have an error of < 3.5% (see Supplementary Table 4). All crossed LJ interactions were calculated with Lorentz–Berthelot rules. Long-range electrostatics employed an implementation of the particle–particle-particle–mesh (PPPM) method^59^. The timestep employed in simulations was 1.1 or 8.9 fs for flexible and rigid nanowindows, respectively. Dimensions of the simulation box in the *x*, *y*, and *z* directions were 42.63 Å, 39.38 Å, and 50.00 Å, respectively. The graphene layer was placed in the *x*–*y* plane. Periodic boundary conditions were employed in *x* and *y* directions. A reflective LJ wall was placed at *z* = 25 Å from the graphene plane.

The graphene framework and nanowindow functional groups were kept flexible in most simulations, except in those with rigid nanowindows and the section of nanowindow dihedral rotation. In the latter, the system was equilibrated with a fully flexible framework at the system temperature. Later, the framework was quenched rigid and its energy minimized followed by another equilibration run and collection.

All molecular models in the figures were prepared using VMD 1.9.3^60^ and rendered with Tachyon^61^.

### Data availability

The data that support the findings of this study are available from the corresponding author F. V.-B. on reasonable request.

## Electronic supplementary material

Supplementary Information

Description of Additional Supplementary Files

Supplementary Movie 1
