# Supplementary material for: Air separation with graphene mediated by nanowindow-rim concerted motion
Source: Nat Commun. 2018 May 4;9:1812. doi: 10.1038/s41467-018-04224-6 (PMC5935753; doi:10.1038/s41467-018-04224-6)
Supplement: Supplementary file 1 — Supplementary Information [file 41467_2018_4224_MOESM1_ESM.pdf]

Supplementary Figures

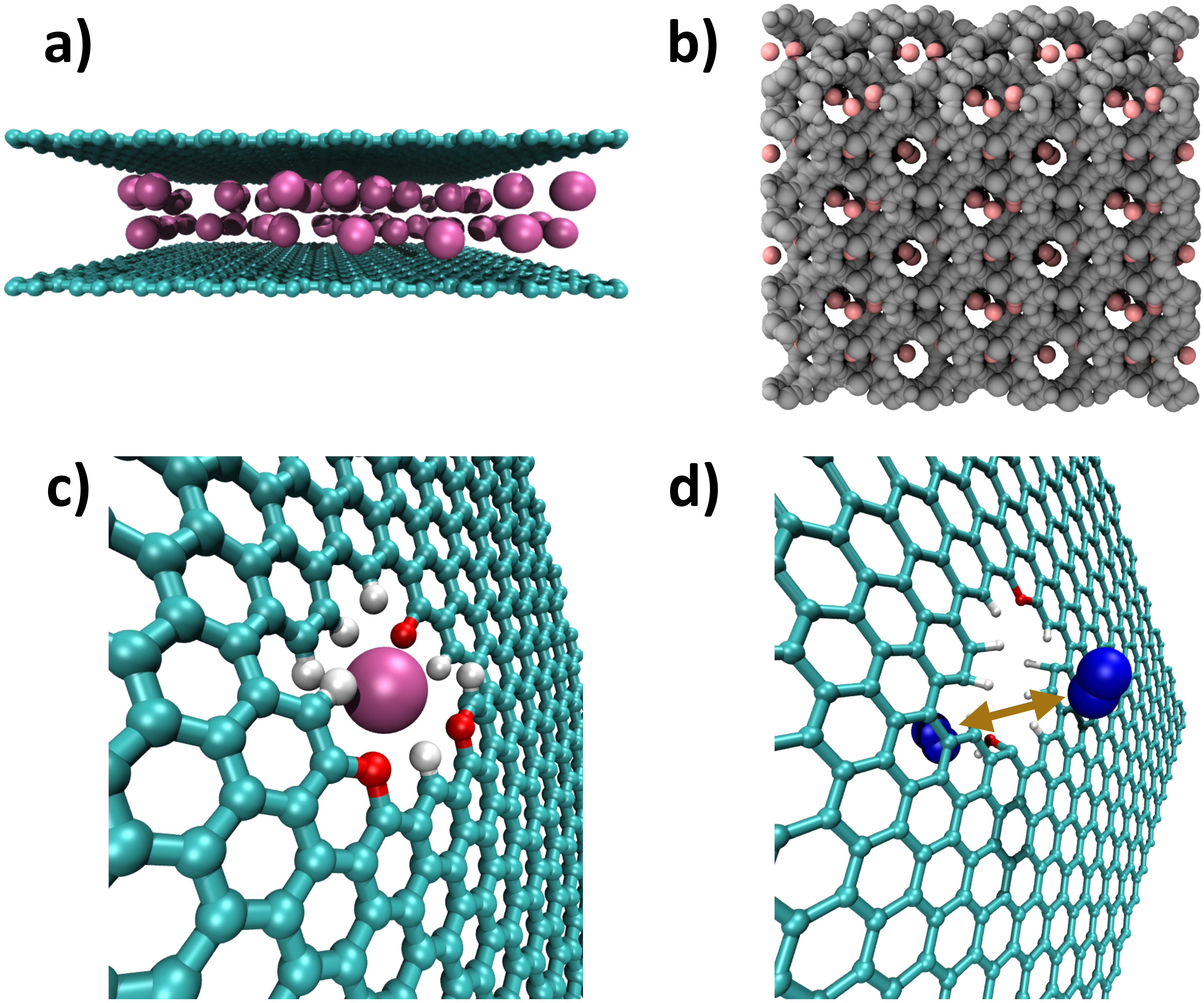

Supplementary Figure 1: Nanopore or nanowindow? We consider the classical definition of nanopore as a nanometer-sized space with a deep potential ready to receive (or adsorb) atoms, for example, **a)** a carbon slit-shaped nanopore with two layer of Ar atoms (purple) adsorbed. **b)** NaX-zeolite nanopore with He atoms adsorbed. Nanowindows are fundamentally different in that **c)** the system energy increases when an atom is inside due to strong repulsion and **d)** due to its single-atom thickness, atoms in one side of the nanowindow can attract atoms of the other side. Nanowindows have the ability to open or close (see Figures 4-5 in the main text) by rotating the functional groups in their rims.

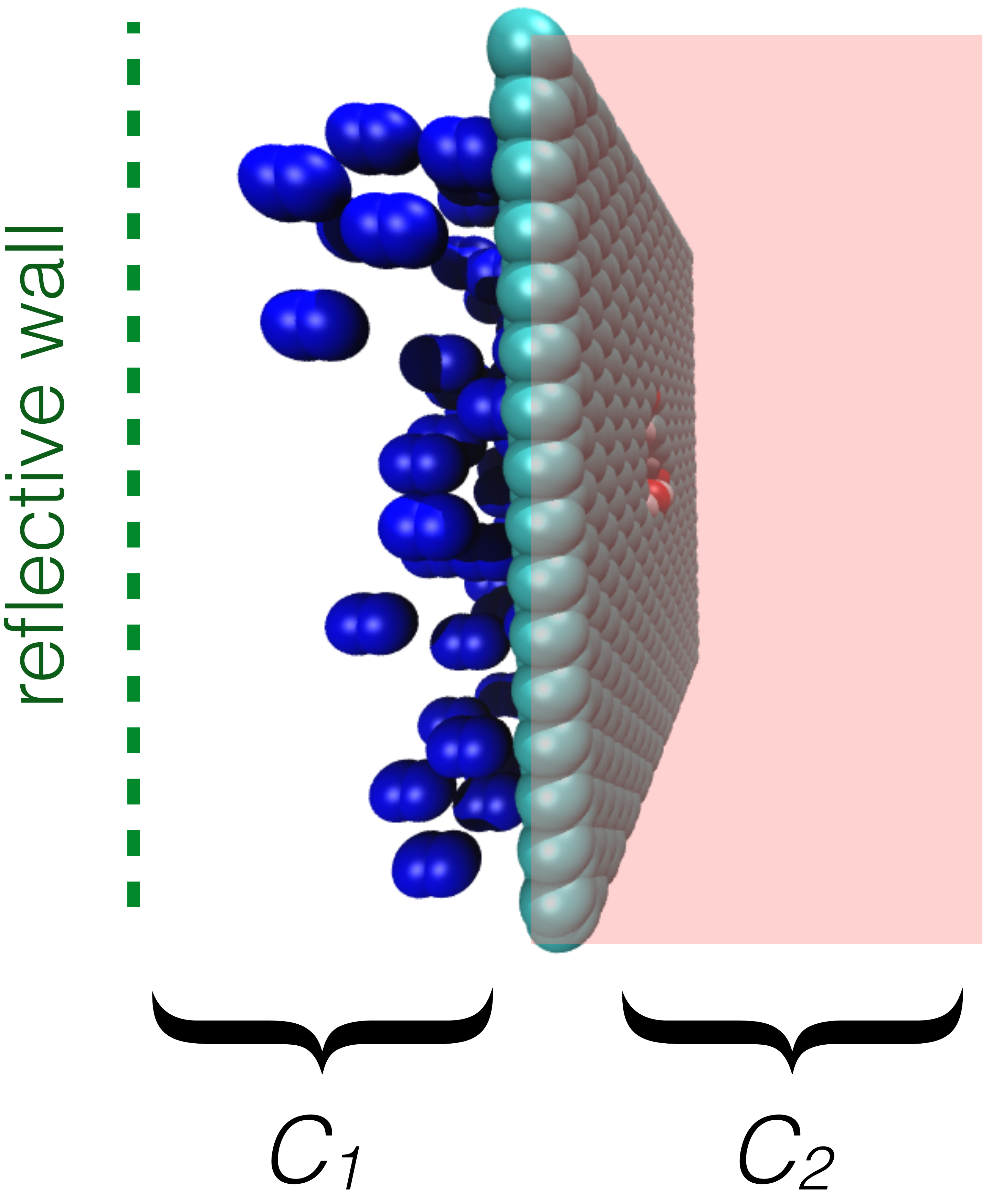

Supplementary Figure 2: Confined system for a material balance on a batch permeation experiment through a nanowindow

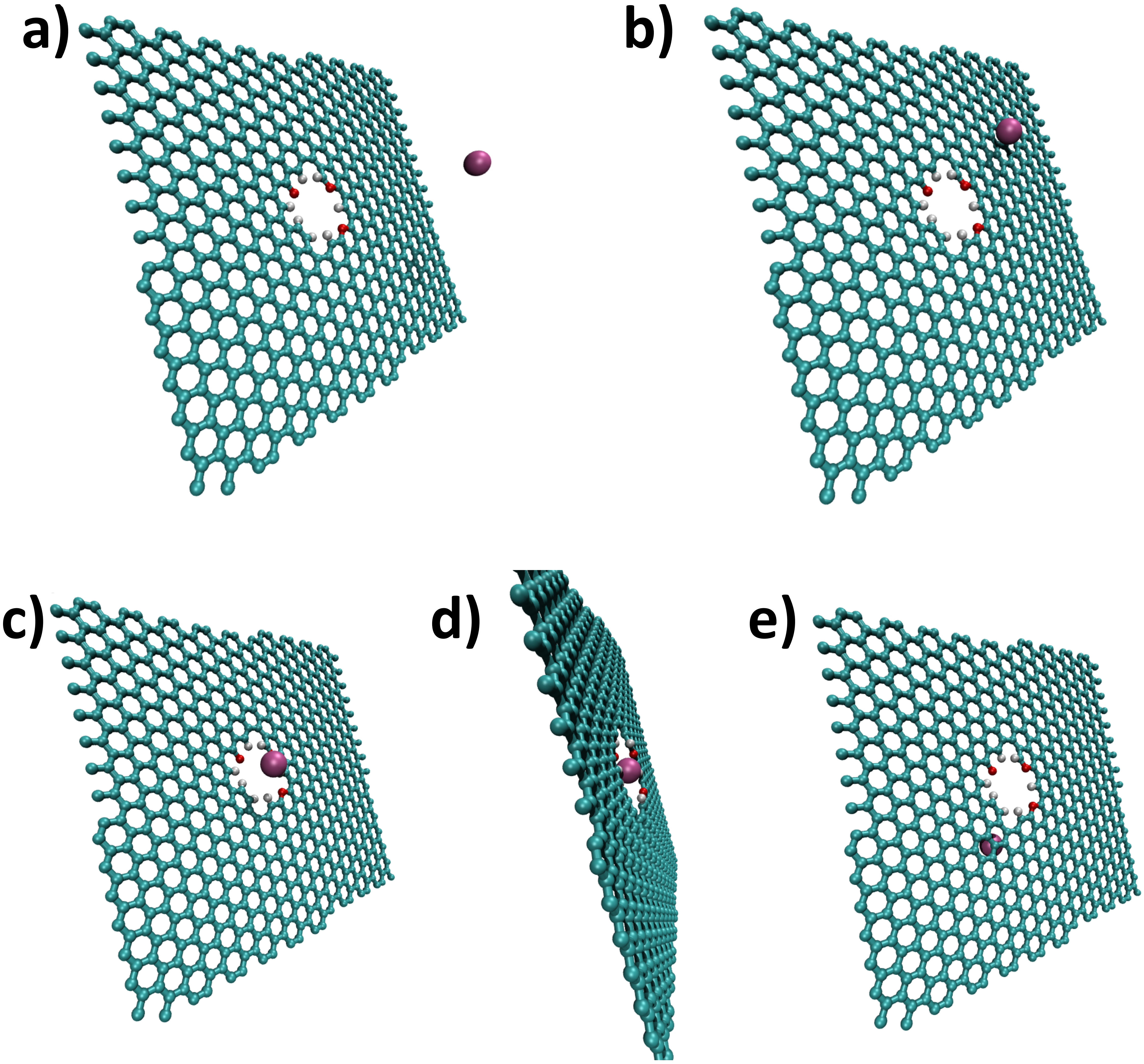

Supplementary Figure 3: Permeation stages for an Ar atom (in purple) through a graphene nanowindow. **a)** Ar atom is in the gas phase and adsorbs in the **b)** the basal plane. **c)** Ar atom left the basal plane and it locates on top of the nanowindow. **d)** Highest energy state occurs when the atom squeezes through the graphene plane. **e)** Successful permeation

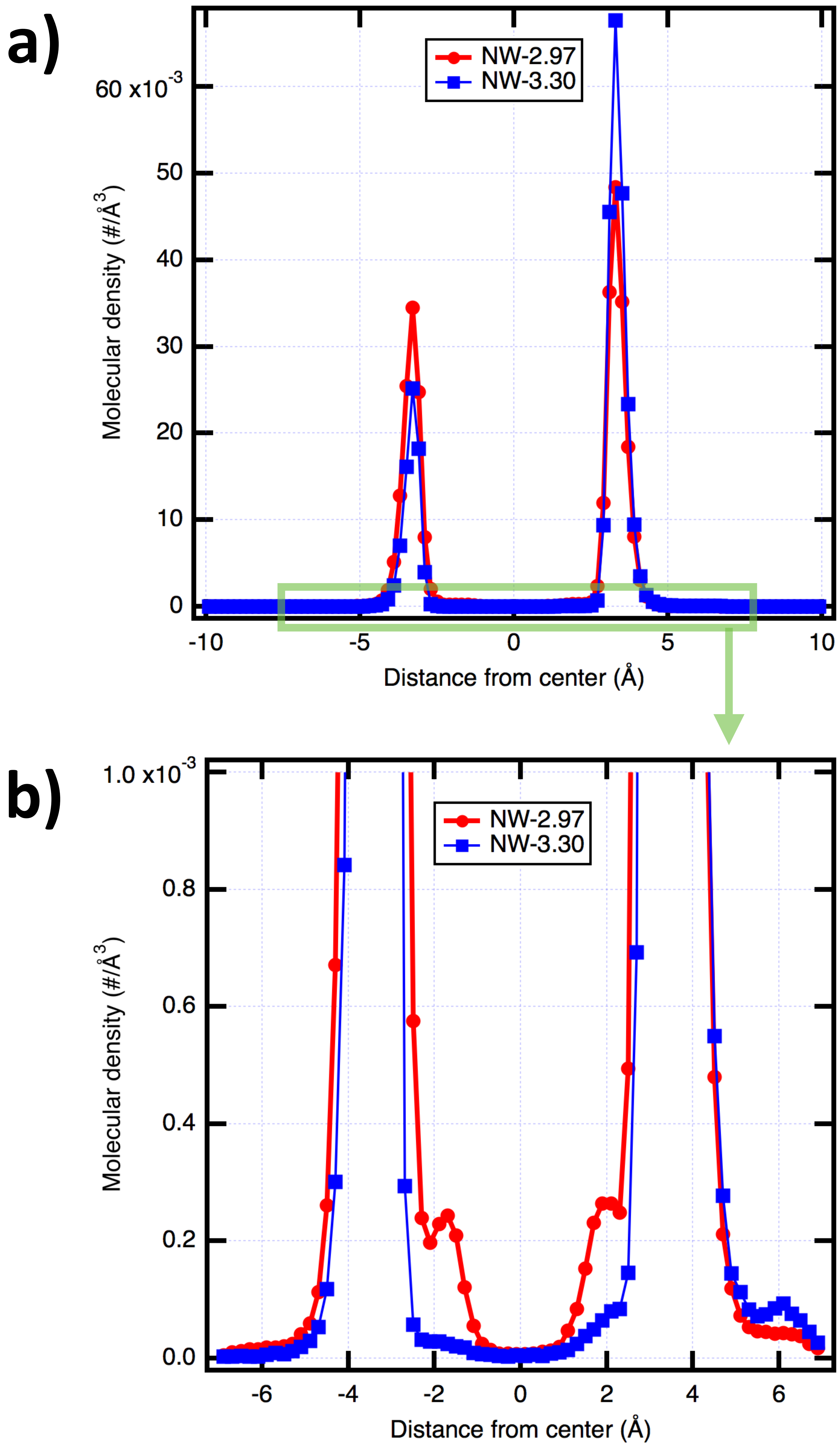

Supplementary Figure 4: **a)** Average density profile of the O_2 center of mass in the plane perpendicular to the graphene layer for NW-2.97 during a molecular dynamics simulation of permeation at 90 K. The two large peaks at ± 3.3 Å represent adsorption on the basal plane of the graphene. **b)** is an enlargement of the region indicated by the green rectangle in **a)** near the center of the nanowindow. The shoulders observed at ± 1.9 Å for NW-2.97 are a consequence of high localization of O_2 molecules of top of this nanowindow.

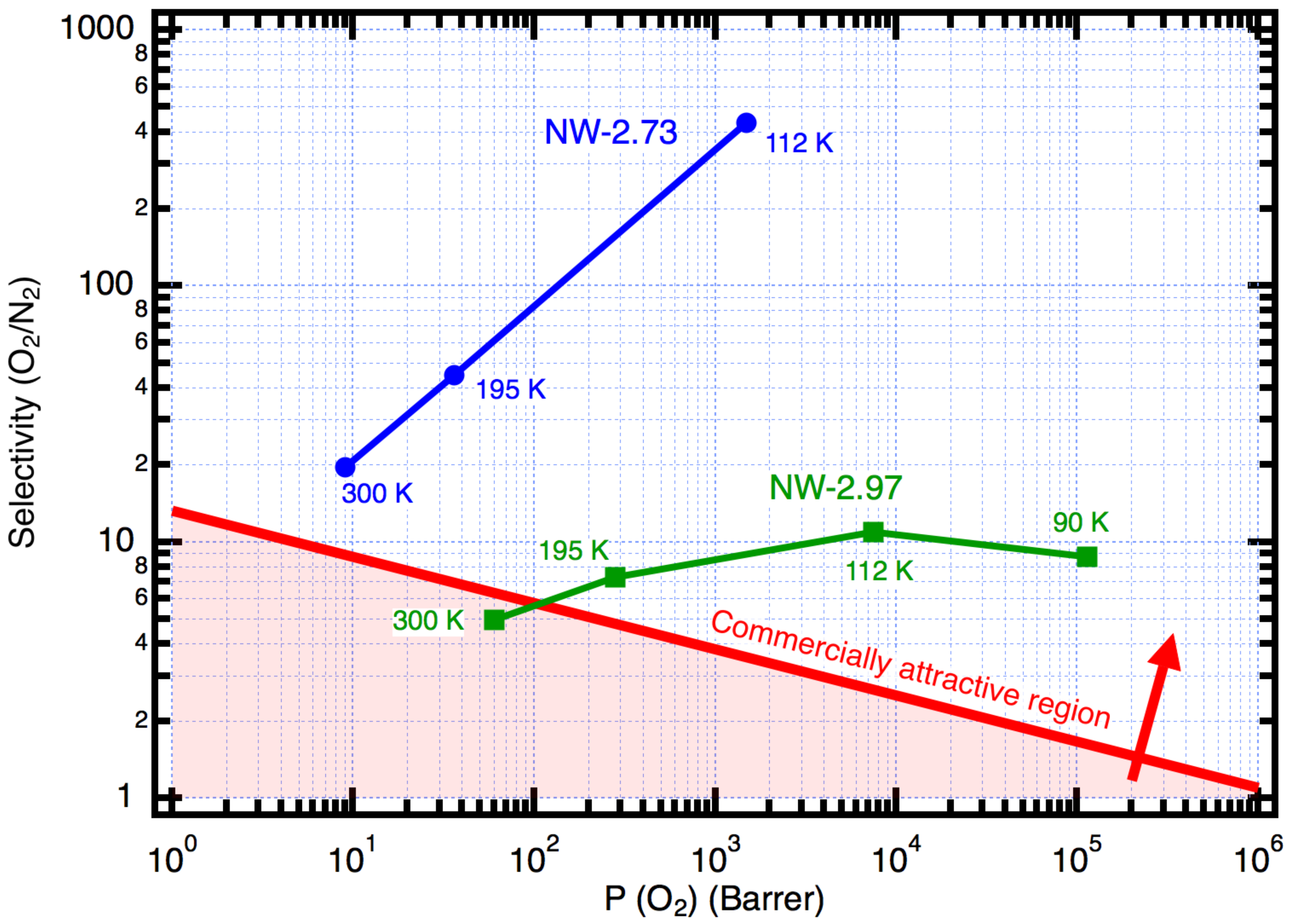

Supplementary Figure 5: Robeson plot showing in pink the location of classical membranes versus our most efficient nanowindows. Low temperature permeation through nanowindow can extend in two orders of magnitude the existing upper bound for air separation. Upper bound limit is based on Robeson's 2008 paper. (1)

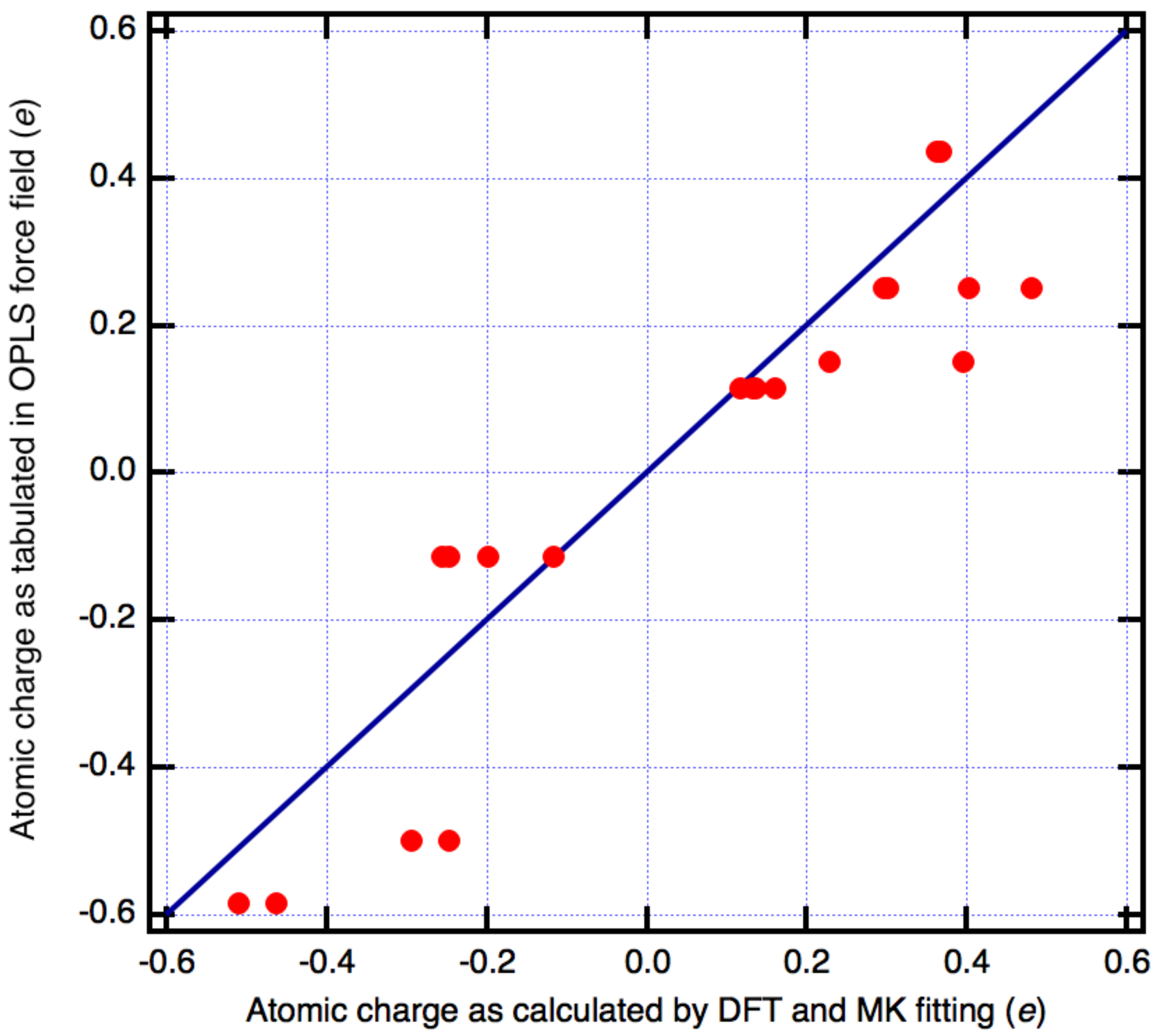

Supplementary Figure 6: Comparison of charges as calculated by MK fitting on DFT optimization on Gaussian09 (2) and charges reported in the OPLS force field employed in molecular dynamics simulations. Blue line shows the space where $q_{MK}=q_{OPLS}$. DFT calculations ran at the B3LYP/6-31G(d) level on a non-periodic graphene-like molecular cluster. Partial atomic charges and electrostatic potentials were estimated through Merz-Singh-Kollman (3) (MK) scheme.

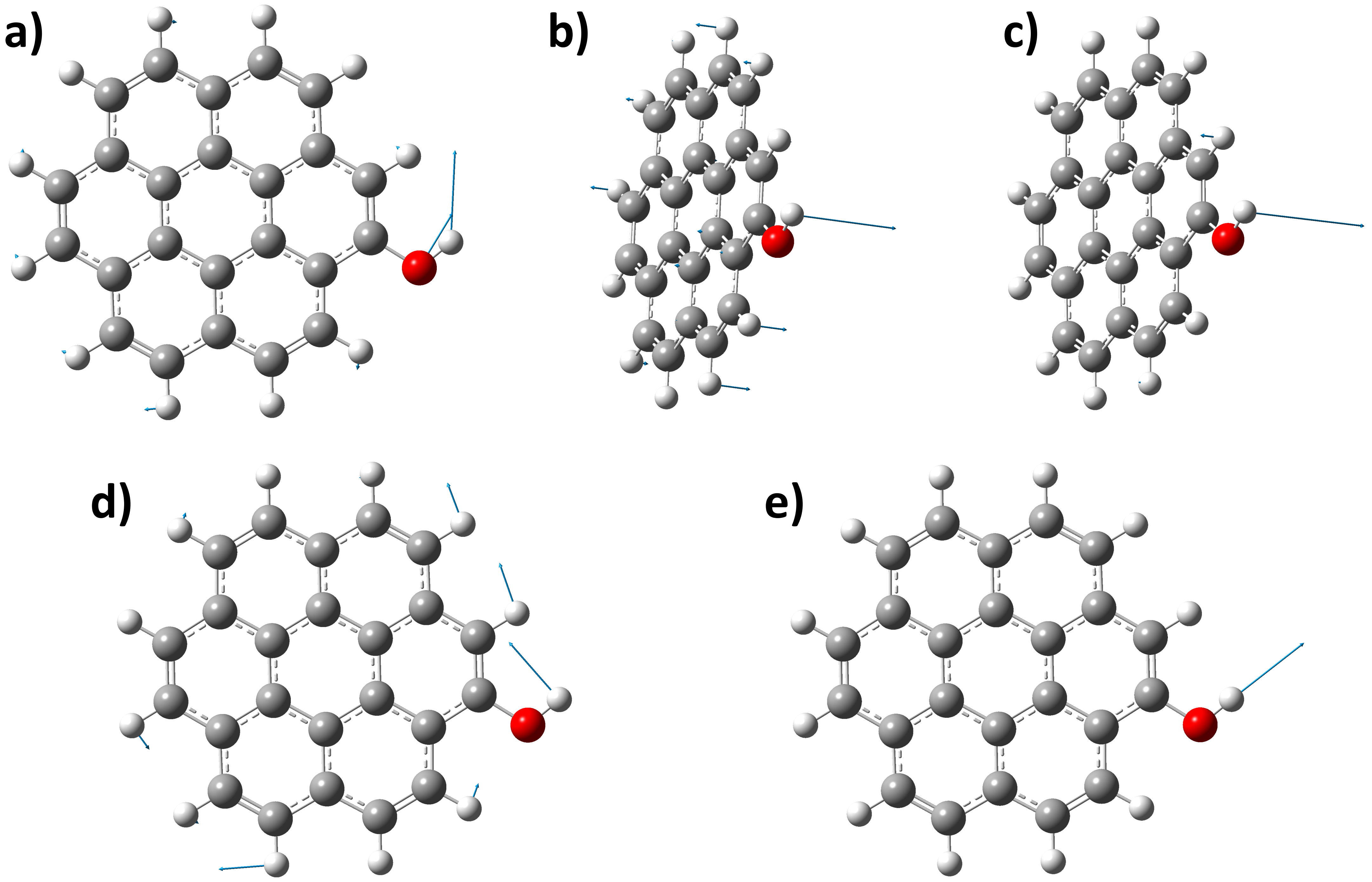

Supplementary Figure 7: Relevant vibrational modes of hydroxy-coronene between high-level DFT* and the molecular mechanics DREIDING force field employed in this work. DFT and DREIDING optimization+frequency calculations ran in Gaussian09 (2). Frequencies scaling factors were 0.989 and 0.936 for DFT (4) and DREIDING (5), respectively.

*APFD on a 6-311+G(2d,p) basis set.

Supplementary Tables

Supplementary Table 1: Functionalization thermochemistry of different graphene nanowindows. $\Delta H_{functionalization*}$ is the energy change from a nanowindow with free carbon edges to a fully functionalized state as shows in Figure 1. These results show that all nanowindows are energetically more stable after functionalization by: (i) an –H termination which is present in most carbon-based materials edges (6), (ii) a phenol –OH termination which is almost ubiquitous in oxidized graphene (7-10). Finally, (iii) ether groups (10,11) which were observed clearly in high resolution STEM–EELS experiments (12). Semiempirical calculations ran in MOPAC2016 (13) at the PM7 (14) level on a periodic graphene framework. Simulation was periodic in the two dimensions of the graphene layer. Periodic translation vectors measure 21.315 and 19.690 Å.

Nanowindow	Functionalization equation	$\Delta H_{functionalization*}$ (kJ mol^{-1})
NW-2.57	$C_{147} + 4.5\ H_2O \rightarrow C_{147}H_9O_2 + 1.25\ O_2$	-2331
NW-2.73	$C_{148} + 5\ H_2O \rightarrow C_{148}H_{10}O + 2\ O_2$	-1714
NW-2.97	$C_{146} + 4\ H_2O \rightarrow C_{146}H_8O_3 + 0.5\ O_2$	-2307
NW-3.30	$C_{138} + 5\ H_2O + 0.5\ O_2 \rightarrow C_{138}H_{10}O_6$	-3304
NW-3.70	$C_{140} + 5\ H_2O \rightarrow C_{140}H_{10}O_4 + 0.5\ O_2$	-2738
NW-3.78	$C_{138} + 5\ H_2O \rightarrow C_{138}H_{10}O_5$	-3143

Supplementary Table 2: DREIDING (15) force field parameters employed in MD simulations for the graphene framework.

name	Parameters	Energy (K)
Bond C-C	r_0=1.421 Å	528450
Bond C-H	r_0=1.090 Å	352300
Bond C-O	r_0=1.420 Å	352300
Bond O-H	r_0=0.98 Å	352300
Angle C-C-C	t_0=120 °	50320
Other angles	t_0=104.51°	50320
Dihedral C-C-C-C	d=-1 n=2	12600
Dihedral C-C-C-O	d=-1 n=2	12600
Dihedral C-C-C-H	d=-1 n=2	12600
Dihedral C-C-O-H	d=-1 n=6	500
Dihedral O-C-C-H	d=-1 n=2	12600
Dihedral H-C-C-C	d=-1 n=2	12600
Dihedral O-C-C-C	d=-1 n=2	12600
Dihedral C-C-O-C	d=-1 n=6	500
Improper C-C-C-C	chi=180°	20100

Supplementary Table 3: Non-bonded force field parameters employed in MD simulations

name	mass ($1.66\ 10^{-27}$ kg)	charge (e)	LJ sigma (Å)	LJ epsilon (K)	reference
C (-*C*=)	12.011	0	3.36	28.0	16
C (*C*-O-H)	12.011	+0.15	3.55	35.2	17
O (C-*O*-H)	15.9994	-0.585	3.07	85.5	17
H (C-O-*H*)	1.00794	+0.435	0	0	17
C (*C*-O-C)	12.011	+0.25	3.80	35.2	17
O (C-*O*-C)	15.9994	-0.50	3.00	59.5	17
C (*C*-H)	12.011	-0.115	3.55	35.2	17
H (C-*H*)	1.00794	+0.115	2.42	15.1	17
Ar	39.948	0	3.405	120.0	16
N (N_2)	14.0067	-0.482	3.31	36.0	18
O (O_2)	15.9994	-0.113	3.02	49.0	18

Supplementary Table 4: Comparison of relevant vibrational modes of hydroxy-coronene between high-level DFT* and the molecular mechanics DREIDING force field employed in this work. DFT and DREIDING optimization+frequency calculations ran in Gaussian09 (2). Frequencies scaling factors were 0.989 and 0.936 for DFT (4) and DREIDING (5), respectively.

	DFT* (cm^{-1})	DREIDING (cm^{-1})	Relative error (%)
a) C–O–H bending	275	318	16
b) –H switching	283	273	-3.5
c) –H switching	314	324	3.2
d) O–H bending	1182	1140	-3.6
e) O–H stretching	3851	2777	-28

*APFD on a 6-311+G(2d,p) basis set.

Supplementary Notes

Supplementary Note 1: Material balance for a batch permeation experiment through a nanowindow depicted in Supplementary Figure 2.

Let the system be the red shaded area of Supplementary Figure 2, initially empty between the graphene layers and the wall. C_2 the number of molecules inside, and C_1 the molecules outside, assuming that rate of permeation is proportional to the number of molecules in each side (first order):

$$\frac{dC_2}{dt} = k_1 C_1 - k_2 C_2$$

where k_1 and k_2 are permeation rate constants.
For simplification, we assume nanowindows to be symmetrical in the graphene plane, then forward and backward permeation rate constants are the same and equal to k. Also , the total amount of particles in the simulation is known (C_T) for the canonical ensemble.

$$k_1 = k_2 = k \qquad\qquad C_T = C_1 + C_2$$

The ordinary differential equation of the system becomes:

$$\frac{dC_2}{dt} = kC_T - 2kC_2$$

After using the initial condition $C_2(t=0)=0$ solves to:

$$C_2(t) = \frac{C_T}{2}\left(1 - \exp\left(-2kt\right)\right)$$

Which linearizes to:

$$\ln\left(1 - \frac{2C_2(t)}{C_T}\right) = -2kt$$

Then, the slope of a plot of the left hand side, against $-2t$ will be the first order permeation constant through a nanowindow.
The constant k has the natural units of frequency.

Supplementary References

(1) Robeson, L.M. *The upper bound revisited*. J. Memb. Sci. 320, 390-400. **(2008)**.

(2) Frisch, M. J.; Trucks, G. W.; Schlegel, H. B.; Scuseria, G. E.; Robb, M. A.; Cheeseman, J. R.; Scalmani, G.; Barone, V.; Mennucci, B.; Petersson, G. A.; et al. *Gaussian–09* Revision D.01.

(3) Singh, U. C.; Kollman, P. A. *An Approach to Computing Electrostatic Charges for Molecules*. Journal of Computational Chemistry, 5 (2), 129–145. **(1984).**

(4) Foresman, J. B.; Frisch, Æ. *Exploring Chemistry with Electronic Structure Methods*, 3rd ed.; Gaussian, Inc.: Wallingford CT, USA, **(1996)**.

(5) *NIST Computational Chemistry Comparison and Benchmark Database*. NIST Standard Reference Database Number 101. Release 18, **(2016)**, Editor: Russell D. Johnson III http://cccbdb.nist.gov/

(6) Berman, D., Deshmukh, S. A., Sankaranarayanan, S. K. R. S., Erdemir, A. & Sumant, A. V. *Extraordinary Macroscale Wear Resistance of One Atom Thick Graphene Layer*. Adv. Funct. Mater. 24, 6640–6646 **(2014)**.

(7) Liu, Z. et al. *Controlling and Formation Mechanism of Oxygen-Containing Groups on Graphite Oxide*. Ind. Eng. Chem. Res. 53, 253–258 **(2014)**.

(8) Dreyer, D. R., Park, S., Bielawski, C. W. & Ruoff, R. S. *The chemistry of graphene oxide*. Chem. Soc. Rev. 39, 228–240 **(2009)**.

(9) Tararan, A., Zobelli, A., Benito, A. M., Maser, W. K. & Stéphan, O. *Revisiting Graphene Oxide Chemistry via Spatially-Resolved Electron Energy Loss Spectroscopy*. Chem. Mater. 28, 3741–3748 **(2016)**.

(10) Bagri, A. et al. *Structural evolution during the reduction of chemically derived graphene oxide*. Nat. Chem. 2, 581–587 **(2010)**.

(11) Yamada, Y. et al. *Subnanometer Vacancy Defects Introduced on Graphene by Oxygen Gas*. J. Am. Chem. Soc. 136, 2232–2235 **(2014)**.

(12) Guo, J. et al. *Crown ethers in graphene*. Nat. Commun. 5, **(2014)**.

(13) Stewart J.J., *MOPAC2016*, Stewart Computational Chemistry, Colorado Springs, CO, USA, http://OpenMOPAC.net **(2016)**.

(14) Stewart, J.J. *Optimization of parameters for semiempirical methods VI: more modifications to the NDDO approximations and re-optimization of parameters*. J Mol Model., 19(1):1-32. **(2013)**.

(15) Mayo, S. L., Olafson, B. D. & Goddard, W. A. *DREIDING: a generic force field for molecular simulations*. J. Phys. Chem. 94, 8897–8909 **(1990)**.

(16) Coasne, B., Jain, S. K., Naamar, L. & Gubbins, K. E. *Freezing of argon in ordered and disordered porous carbon*. Phys. Rev. B 76, 85416 **(2007)**.

(17) Jorgensen, W. L., Maxwell, D. S. & Tirado-Rives, J. *Development and Testing of the OPLS All-Atom Force Field on Conformational Energetics and Properties of Organic Liquids*. J. Am. Chem. Soc. 118, 11225–11236 **(1996)**.

(18) Potoff, J. J. & Siepmann, J. I. *Vapor–liquid equilibria of mixtures containing alkanes, carbon dioxide, and nitrogen*. AIChE J. 47, 1676–1682 **(2001)**.
