# Supplementary material for: Air separation with graphene mediated by nanowindow-rim concerted motion
Source: Nat Commun. 2018 May 4;9:1812. doi: 10.1038/s41467-018-04224-6 (PMC5935753; doi:10.1038/s41467-018-04224-6)
Supplement: Supplementary file 2 — Description of Additional Supplementary Files [file 41467_2018_4224_MOESM2_ESM.docx]

**Description of Additional Supplementary Files**

File Name: Supplementary Movie 1

Description: Animated molecular dynamics snapshots for a nanowindow with N_2_ adsorbed. Dynamic vibrations of the nanowindow rim are evident. Cyan, white, red and blue atoms are C, H, O and N, respectively.
